# The value of ultrasound-guided biopsy of fluorodeoxy-glucose positron emission tomography (FDG-PET)-positive supraclavicular lymph nodes in patients with suspected lung cancer

**DOI:** 10.1186/s12880-017-0214-8

**Published:** 2017-07-11

**Authors:** Lennart Werner, Franziska Aebersold Keller, Ujwal Bhure, Justus Egidius Roos, Katharina Tornquist, Maria del Sol Pèrez-Lago, Oliver Gautschi, Klaus Strobel

**Affiliations:** 1 Department of Radiology and Nuclear Medicine, Cantonal Hospital, Spitalstrasse 13, 6000 Lucerne, Switzerland; 2 Department of Pathology, Cantonal Hospital, Lucerne, Switzerland; 3 Department of Medical Oncology, Cantonal Hospital, Lucerne, Switzerland

**Keywords:** NSCLC, PET/CT, Supraclavicular lymph nodes, US guided biopsy, Staging

## Abstract

**Background:**

Accurate lymph node staging is essential for adequate prognostication and therapy planning in patients with non-small cell lung cancer (NSCLC). FDG-PET/CT is a sensitive tool for the detection of metastases, including non-palpable supraclavicular lymph node (SCLN) metastases. Histological proof of metastatic spread and mutation analysis is crucial for optimal staging and therapy. The aim of this study was to investigate the value of ultrasound-guided fine needle aspiration cytology (FNAC) and core biopsy (CB) of FDG active, non-palpable SCLN’s in patients with suspicion for lung cancer.

**Methods:**

Twelve consecutive patients with suspected lung cancer and FDG-positive SCLN underwent FNAC (*n* = 11) and/or CB (*n* = 10) and were included and evaluated retrospectively in this study. Cytologic and/or histologic evaluation was performed to confirm initially suspected diagnosis (lung cancer), to confirm N3 stage, and to screen for driver mutations in lung adenocarcinoma.

**Results:**

FNAC alone showed diagnostic success in 11/11 cases (100%), CB alone in 9/10 patients (90%), and the combination of both procedures was successful in 12/12 cases (100%). Lymph node metastases from NSCLC (7 adenocarcinoma, 2 squamous cell carcinoma) could be confirmed in 9 patients. Other diagnoses were small cell lung cancer (SCLC), breast cancer and sarcoidosis. There was enough material for immunhistochemistry in all patients. For molecular testing, material from this lymph node biopsies and lung biopsy was used. In two patients with adenocarcinoma of the lung driver mutations were detected (EGFR Exon 19 deletion and ALK rearrangement) out of the lymph node metastasis.

**Conclusions:**

US-guided combined FNAC and CB of FDG positive supraclavicular lymph nodes in patients with suspected lung cancer is a safe and effective procedure to confirm N3-stage and to obtain representative material for molecular testing.

## Background

The diagnosis of supraclavicular lymph node (SCLN) metastases in non-small cell lung cancer (NSCLC) indicates N3 and at least stage IIIB disease and results usually in a non-surgical approach, or even a palliative treatment. Open surgery of SCLN is invasive and might be difficult in small metastatic and non-palpable lymph nodes and is associated with the risk of vascular injuries. FDG-PET/CT enables the visualization of SCLN metastases at a low detection limit of 3–4 mm on the basis of increased FDG uptake [1, 2]. If FDG active SCLN metastases are suspected in PET/CT, US guided biopsy might serve as a minor invasive, clinically very important approach, to confirm metastatic spread and obtain tissue for mutation analysis, especially if no other distant metastases are present. US guided fine needle aspiration cytology (FNAC) of SCLN has been successfully used for years [1, 3–6]. In the last years, major clinical progress was achieved with the identification of oncogenic driver mutations in tumor tissue followed by targeted systemic therapies in patients with advanced lung adenocarcinoma [7–10]. Thus, US-guided SCLN biopsy might be very useful for the decision of optimal treatment.

The aim of this study was to investigate the feasibility, safety and the value of ultrasound-guided fine needle aspiration cytology (FNAC) and core biopsy (CB) of FDG active, non-palpable SCLN’s in patients with suspicion for lung cancer.

## Methods

Since June 2012 we routinely implemented to use ultrasound guided core and fine needle biospy for further evaluation of FDG positive non-palpable supraclavicular lymph nodes in patients with suspicion for lung cancer. Before June 2012 suspicious supraclavicular lymph nodes were resected operatively, if palpable, and otherwise were not biopsied at all. Retrospectively, 12 patients (8 male, 4 female) with a mean age of 64,3 years (range 46 - 79 years) with suspicion for lung cancer and FDG active SCN followed by ultrasound guided biopsy between June 2012 and March 2014 were identified by a systematic search in the institutional PET/CT database and included in this study. Four patients had a history of tumors (2 breast cancer, 1 melanoma, 1 urothelial carcinoma of the urethra) other than lung cancer and one patient had two other cancers (prostate and urothelial carcinoma of the urinary bladder). Positive approval of ethics committee was obtained for this study.

In all patients a partial body (skull until midthigh) FDG-PET/CT was performed 60 min. After intraveneous injection of approx. 300 (range 270-330) MBq F18-FDG (Discovery 600, GE Healthcare). Diagnostic chest CT with intraveneous contrast was also available in all patients or added during PET/CT.

In all patients FDG-PET/CT was performed and analyzed in the Picture Archiving and Communication System (PACS) by a doubly board certified radiologist and nuclear medicine physician with 12 years experience in reading PET/CT examinations in tumor patients regarding the presence of a primary lung tumor, lymph node and distant metastases. Supraclavicular lymph nodes of any size with increased FDG uptake (higher than mediastinal background) were diagnosed as suspicious. In all patients ultrasound guided FNAC or CB of PET positive SCLN were performed by a radiologist with 20 years experience in ultrasound guided biopsies.

The patients were placed in supine position. A 5-12 Mhz linear probe (IU 22 ultrasound device, Philips Healthcare) was used to localize the FDG avid supraclavicular lymph nodes. Transverse and sagittal images were obtained and largest diameter of SCLN were documented. US-guided FNAC was performed after local anesthesia under ultrasound guidance with a 22 gauge needle and aspriated cells were scretched out immediately on a glass slide. At least two consecutive fine needle biopsies were performed. 16 gauge biopsy needles (Temno Evolution, Carefusion, IL, USA) were used for core biopsies. Cytologic smears and histologic samples were analyzed for the presence of tumor cells/tissue by an experienced pathologist. Additionally mutation analysis were performed for various mutations (EGFR, KRAS, ALK).

## Results

**Fig. 1:**
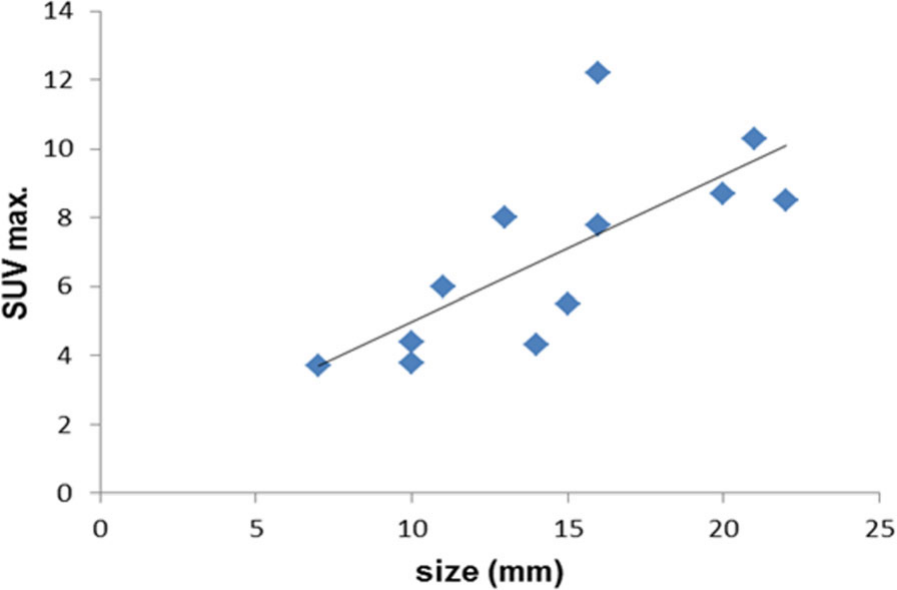
The relationship between SCLN size and SUV max (*r* = 0.73, *p* = <.05)

**Fig. 2:**
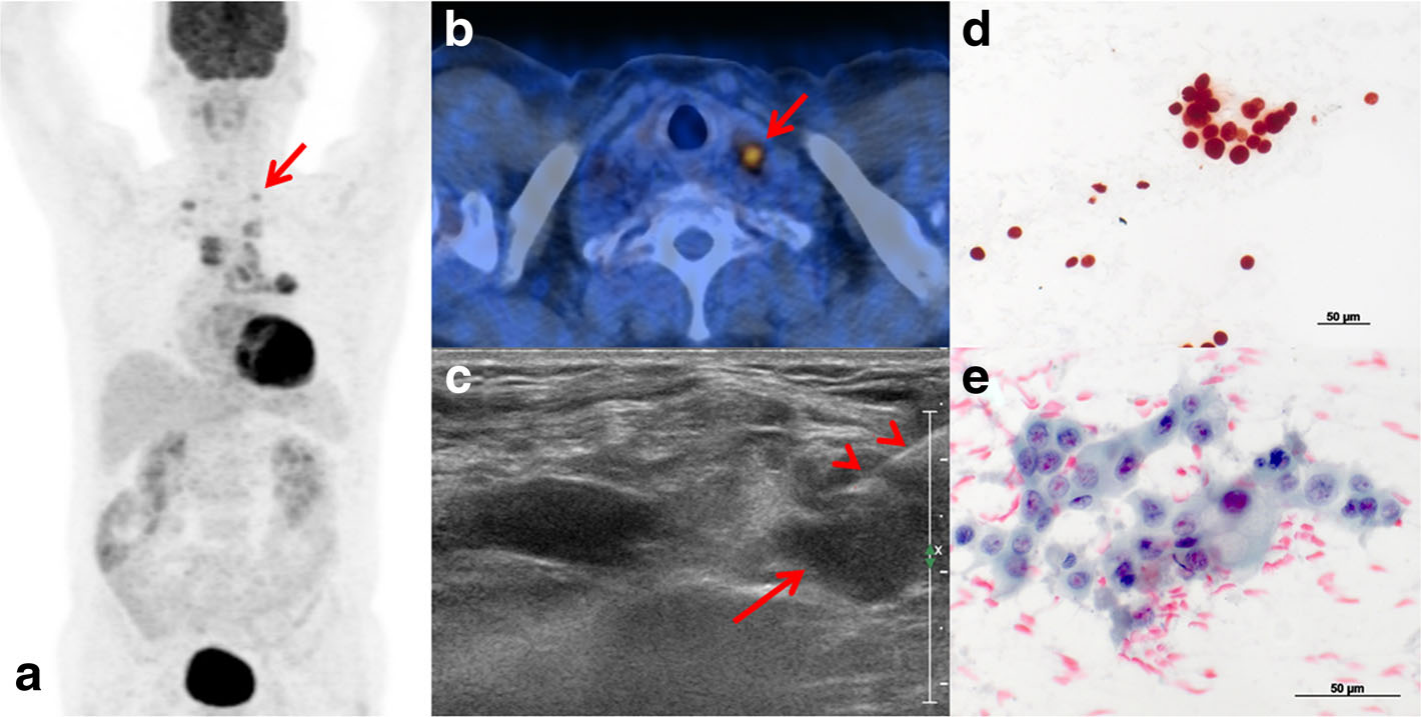
FDG PET/CT of a patient with suspected lung cancer. Bronchoscopy performed in an external hospital was not diagnostic. MIP image **a** shows a primary tumor in the left lung with bilateral FDG active mediastinal and supraclavicular lymph nodes (*arrow*). Axial fused PET/CT **b** and ultrasound **c** showed a SCLN (*arrow*) which was biopsied with fine and core needle (*arrowheads*). Cytology showed TTF 1 positive **d** adenocarcinoma (**e**). In this case no driver mutation identified

**Fig. 3:**
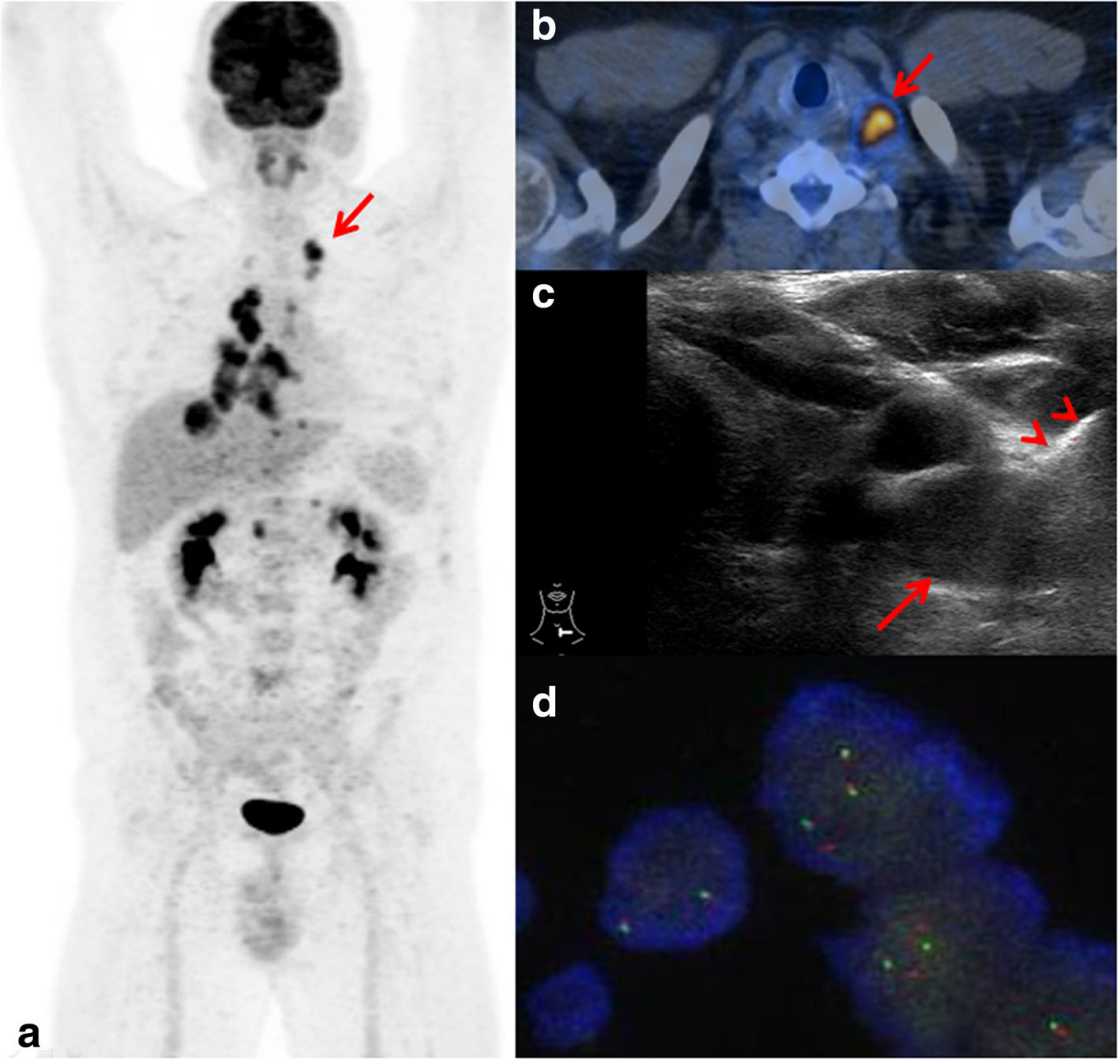
FDG PET/CT of a patient with suspected metastatic lung cancer. Maximum intensitiy projection image **a** showed primary lung cancer in the right lower lobe with extensive hilar, mediastinal, bilateral supraclavicular (*arrow*) and abdominal lymph node metastases. Small bone metastases e.g. in the left iliac bone with low metabolic activity were visible. In MR (not shown) multiple brain metastases were detected. Fused axial PET/CT image **b** showed FDG active left sided SCLN (*arrow*). Ultrasound guided FNAC and CB documentation (*arrowheads*) (**c**). Molecular testing showed ALK rearrangement **d** and the patient was treated with brain radiation and crizotinib

Bilateral involvement of SCLN’s was present in 3/12 (25%) patients, unilateral in the remaining 9/12 (75%) patients. All patients with FDG active SCLN’s had additional FDG active hilar and mediastinal lymph nodes. Six patients had also distant metastases visible on PET/CT. In all patients the FDG active SCLN’s were visible with US. The lymph nodes chosen for US-guided FNAC/CB had a mean size of 14.6 mm (range 7–22 mm) and a mean maximum standard uptake value (SUVmax.) of 6.9 (range 3.7–12.2). The mild linear positive correlation between lymph node size and SUV max. was seen as shown in Fig. 1 (*r* = 0.73, *p* = <.05). Lymph node metastases from NSCLC (7 adenocarcinoma, 2 squamous cell carcinoma) could be confirmed in 9 patients (Fig. 2). Other diagnoses were SCLN metastasis of SCLC and breast cancer. One patient had sarcoidosis mimicking a lung cancer with lymph node metastases. FNAC alone showed diagnostic success in 11/11 cases (100%), CB alone in 9/10 patients (90%), and the combination of both procedures was succesful in 12/12 cases (100%). We observed no complications caused by the ultrasound biopsies. Immunochemistry and molecular testing for driver mutations was possible in most of the patients. In two patients with adenocarcinoma of the lung, actionable driver mutations were detected in the lymph node tissue, leading to systemic targeted therapies. (Fig. 3). In two others, material from the primary tumor showed another two mutations (KRAS p.G12 V mutation and KIF5B-Ret translocation). The patient with KRAS mutation refused a targeted therapy, the patient with the RET translocation was treated with vandetanib and cabozantinib. On patient had a bronchoscopy before US guided SCLN biopsy, which was not diagnostic and planned endobronchial ultrasound (EBUS) was no longer needed after successful SCLN biopsy.In 6 (50%) patients only SCLN biopsy was performed without the need for other invasive interventions. Results and details recording mutations and treatment are summarized in Table 1.

**Table 1:** Characteristic of the patients

Nr	size of SCLN in mm	FNAC	core biopsy	diagnosis from SCLN biopsy	immunhistochemistry	molecular testing for driver mutations	tumor history besides suspicion for lung cancer	distant metastases	other biopsies	targeted theraphy
1	15	diagnostic	diagnostic	NSCLC (adenoca)	CK +, TTF1 +, Napsin A+	negative	none	none	bronchoscopic biopsy before PET/CT not diagnostic	
2	16	diagnostic	not performed	SCLC	CD56 +,TTF1 -,mib1 > 90%	not performed	none	liver	none	
3	21	diagnostic	diagnostic	NSCLC (adenoca)	TTF1 +, BerEP4 +	ALK Rearrengement (confirmation in brain metastasis)	none	bone, brain, abdominal In	not enough material from bronchoscopic biopsy for mutation analysis,	crizotinib
4	20	diagnostic	diagnostic	NSCLC (squamous cell ca.)	p63 +, CK5/6 +, CK7-, CK20-, TTF1 -, mib1 50-80%	negative	none	abdominal In, lung, kidney	SCLN biopsy and later bronchoscopic biopsy performed	
5	22	not performed	diagnostic	NSCLC (adenoca)	CK7 +, TT1 -, Napsin A -, p63 -, CK 5/6 -, BerEP4 +	negative	urothelca, urethra	bone, adrenal, abdominal In	none	
6	16	diagnostic	diagnostic	NSCLC (squamous cell ca.)	p63 +, CK5/& +, TTF1 -, CK7 (+)	negative	none	pleura	broncho positive	
7	10	diagnostic	not performed	NSCLC (adenoca)	TTF1 +	lung biopsy: pF1 2 V KRAS	none	none	none	
8	13	diagnostic	diagnostic	NSCLC (adenoca)	TTF1 +, CK5/6 -	lung lobectomy: KIF5B-RET Translokation	malignant melanoma	lung, abdominal In	none	vandetanib, cabozantinib
9	14	diagnostic	diagnostic	granulomas (sarcoidosis)	not performed	not performed	breast cancer	none	none	
10	11	diagnostic	not diagnostic	NSCLC (adenoca)	TTF1 +	negative	none	none	none	
11	7	diagnostic	diagnostic	breast cancer (adenoca.)	CK7 +, BRST +, TTF1 -	negative	breast cancer, bilateral lung cancer	none	CT biopsy lung	
12	10	diagnostic	diagnostic	NSCLC (adenoca)	CK7+, TTF1+	Del p.E746_A750del in Exon 19 von EGFR	prostate camcer, urothelca, urinary	none	none	afatinib

*abbreviations*: *NSCLC*, non-small cell lung cancer, *SCLC* small cell lung cancer, *ln* lymph node, *FNAC* fine needle aspiration cytology, *SCLN* supraclavicular lymph node

## Discussion

This study shows that US guided fine needle biopsy and CB are safe and successful in the majority of NSCLC patients with FDG active SCLN’s. The interventions are also feasible in small lymph nodes (<2 cm) – the smallest successfully biopsied lymph node measured 7 mm in our series. With this approach, more invasive and costly procedures to obtain tumor tissue and to prove N3 disease like bronchoscopy, open lymph node biopsy, mediastinoscopy or CT guided lung biopsy, can be avoided in many patients. 50% of our study patients needed no biopsy of the primary tumor, because the diagnosis was established by investigation of SCLN’s alone. Several authors reported the value of US and FNAC of SCLN’s in lung cancer patients by showing that US is more sensitive than palpation and CT [1, 3, 6, 11]. PET/CT is the currently the imaging method of choice for staging of lung cancer patients [12, 13]. We could show that PET/CT has a high positive predictive value for SCLN metastases in lung cancer patients using US guided biopsy as reference. If increased uptake is visible on PET/CT scans US guided biopsy should be considered despite the SUVmax. value.

In the age of personalized therapy for lung cancer it is crucial to obtain representative and sufficient tumor tissue for molecular testing, especially in lung adenocarcinoma [10]. We could show that biopsy of SCLN’s may provide representative tumor cytology and histology for molecular testing. In two of our 12 patients, actionable driver mutations could be identified by SCLN biopsy and targeted therapy was initiated. US-guided core neck biospies are safe and in a previous study no clinical relevant complications were described even in patients with antiplatelet/anticoagulation therapy [14]. Although we observed no procedure related complications in our patients, core biopsy should only be performed by physicians which are well trained in imaging guided interventions because relevant complications like carotid injury and hematoma causing tracheal compression with dyspnoe have been reported in ultrasound guided neck biopsies [15, 16].

## Conclusions

US-guided combined FNAC and CB of FDG positive supraclavicular lymph nodes in patients with suspected lung cancer is a safe and effective procedure to confirm N3-stage and to obtain representative material for molecular testing.

## Abbreviations

CB: core biopsy
EBUS: endobronchial ultrasound
EGFR: epidermal growth factor receptor
FDG: fluorodesoxyglucose
FNAC: fine needle aspiration cytology
NSCLC: non small cell lung cancer
PET/CT: positron emission tomography/computer tomography
SCLC: small cell lung cancer
SCLN: supraclavicular lymph node
US: ultrasound
